# Trends in acute care of cerebrovascular events – a joinpoint analysis with German hospital data from 2000 to 2021

**DOI:** 10.1186/s42466-025-00404-0

**Published:** 2025-06-27

**Authors:** Richard Schmidt, Charlotte Huber, Johann Otto Pelz, Joseph Classen, Dominik Michalski

**Affiliations:** 1 Department of Neurology, Medical Faculty, Leipzig University, Leipzig, Germany; 2 Institute for General Practice and Family Medicine, University Hospital Jena, Friedrich Schiller University Jena, Jena, Germany

**Keywords:** Stroke, Acute stroke care, Stroke unit, Time trends, Administrative data, Mortality

## Abstract

**Background:**

Acute stroke care has evolved markedly in recent decades, yet long-term trends across stroke subtypes remain understudied. This study analyzed national trends in inpatient stroke care for ischemic stroke (IS), intracerebral hemorrhage (ICH), and subarachnoid hemorrhage (SAH) in Germany from 2000 to 2021.

**Methods:**

We conducted a retrospective analysis of nationwide hospital administrative data, assessing annual case counts, age-standardized rates, mean length of stay, and annual inpatient case days (AICD). Stroke unit (SU) treatments were analyzed from 2005 onward. Joinpoint regression identified changes in trends over time.

**Results:**

IS case rates, length of stay, and AICD declined significantly until 2005/2006, after which they stabilized at remarkably high levels. Paralleled by a rapid expansion of SU care, in-hospital mortality from IS decreased significantly. Coding of unspecified stroke (I64) declined steeply, suggesting shifts in diagnostic precision. In contrast, ICH and SAH showed falling case rates but increasing lengths of stay, particularly among deceased patients. SU treatments rose continuously from 2005 to 2021, with age-standardized rates increasing by 7.1% annually.

**Conclusions:**

Over two decades, total inpatient burden from stroke has declined, primarily due to reductions in IS admissions and mortality. However, longer hospital stays in SAH and ICH and an overall rising SU care indicate shifting but consistently high resource requirements. Thus, continued efforts in optimizing healthcare infrastructure seem reasonable and should consider a subtype-specific resource allocation in acute stroke care.

## Introduction

Stroke remains a leading cause of morbidity and mortality worldwide, affecting over 101 million individuals, with approximately 12 million new cases annually [1–4]. Despite progress in prevention, acute therapy, the absolute number of cases continues to rise due to demographic shifts [5–8]. Acute stroke care increasingly involves subtype-specific treatments: ischemic stroke (IS) management focuses on vessel recanalization via intravenous thrombolysis and endovascular intervention [9]; intracerebral hemorrhage (ICH) treatment prioritizes blood pressure control, hemostatic management, and surgery in selected cases [10]; and subarachnoid hemorrhage (SAH) often requires immediate intensive care with surgical or endovascular procedures [11].

Understanding long-term trends in the utilization of stroke care infrastructure is essential for resource allocation [12]. In this context, especially stroke units (SUs), representing specialized hospital units for acute stroke management, have gained attention since improved outcomes were shown through standardized protocols and an optimized infrastructure [13, 14]. However, comprehensive analyses of temporal changes in institutional stroke care, particularly regarding the use of SUs, remain limited.

We aimed to close this study gap by analyzing hospitalized stroke cases in Germany from 2000 to 2021, stratified by subtype (SAH, ICH, and IS), and SU utilization from 2005 onward. Using routinely collected hospital administrative data, we provide a statistically agnostic, data-driven perspective on evolving stroke care patterns to facilitate future healthcare planning [15, 16].

## Methods

### Data sources

This study utilizes aggregated, anonymized hospital administrative data provided by the Federal Statistical Office of Germany, based on mandatory reporting under the Hospital Statistics Regulation (KHStatV) and the Federal Statistics Act (BStatG). Data include all inpatient cases with a primary diagnosis of SAH (ICD-10 I60), ICH (ICD-10 I61), IS (ICD-10 I63), and stroke not specified as hemorrhage or infarction (ICD-10 I64) from 2000 to 2021 [17]. Due to prior validation studies indicating high sensitivity for capturing IS when using ICD-10 I63 and I64 [18–20], both codes were combined under the IS category.

For the trend analysis of stroke subtypes, we included all inpatient cases with a documented residence in Germany, aged 20 to 89 years, who were hospitalized with a primary diagnosis of SAH (ICD-10 I60), ICH (ICD-10 I61), IS (ICD-10 I63 or I64), between 2000 and 2021. Cases with unknown or foreign place of residence, as well as patients under 20 or over 89 years of age, were excluded. Age was categorized in five-year bands.

Additionally, hospital case-related performance data are reported under the Hospital Remuneration Act (KHEntgG) using the German Operation and Procedure Classification (OPS). Since 2005, specialized SUs treatment has been encoded under OPS 8-981 [21], which is used as a sensitive surrogate for high-quality acute stroke care [22, 23]. We included all inpatient cases from 2005 to 2021 with documented treatment in a certified stroke unit, identified by OPS code 8-981, regardless of primary diagnosis. As with the dataset of stroke subtypes, inclusion was limited to patients aged 20 to 89 years with a documented residence in one of the 16 German federal states. Cases without OPS 8-981 coding were excluded to ensure comparability and capture only acute stroke unit care [24].

As this study uses routinely collected, anonymized data, individual informed consent was not required. The institutional review board of the Medical Faculty of Leipzig University reviewed the study and exempted it from ethics approval. Reporting adheres to the RECORD statement guidelines [25].

### Preprocessing and standardization

Annual absolute counts of discharged and deceased cases per diagnosis, as well as cases treated in stroke units (SUs), were extracted. Age- and sex-standardized rates per 100,000 population were calculated using German census data and the 2013 European standard population [26, 27]. Confidence intervals (95%-CI) were estimated using Byar’s method with Dobson adjustment, implemented via the PHEindicatormethods R package [28].

Weighted mean length of hospital stay was derived from age- and sex-specific data. To assess overall demand for acute stroke care, the annual inpatient case days (AICD) were computed as the product of weighted mean length of stay and absolute case counts.

### Joinpoint regression for trend analyses

Temporal trends were analyzed using Joinpoint Regression (JPR) [29]. The Joinpoint software provided by the National Cancer Institute detects statistically significant changes in trends (joinpoints) and estimates annual percent change (APC) between joinpoints, along with the average annual percent change (AAPC) for the entire study period [30]. Models were constructed for absolute counts, standardized rates, mean length of stay, and AICD for each stroke subtype, as well as for SU-treated cases. To prevent overfitting, a maximum of four joinpoints was permitted, with model selection based on the Weighted Bayesian Information Criterion. Statistical significance was set at *p* < 0.05.

All calculations were performed with R Statistical Software (Version 4.3.1) in R Studio (Version 2023.09.0 + 463) [31, 32].

## Results

AAPCs for the period 2000–2021 are summarized in Table 1. Across all stroke subtypes, accumulated annual inpatient case days (AICD) significantly decreased in both discharged (AAPC = -1.0, 95%-CI -1.6 to -0.4, *p* = 0.001) and deceased cases (AAPC = -1.2, 95%-CI -2.0 to -0.3, *p* = 0.006).

**Table 1:** Annual average percent change of stroke subtypes (2000–2021) and cases treated on stroke unit (2005–2021), Germany

Category	Measure	Discharged			Deceased		
		AAPC	Lower CI	Upper CI	AAPC	Lower CI	Upper CI
Subarachnoid Hemorrhage	Case Count	**-1.3**	**-1.8**	**-0.8**	-0.6	-1.4	0.1
	Case Rate	**-1.9**	**-2.4**	**-1.5**	**-1.4**	**-2.3**	**-0.6**
	Length of Stay	**1.6**	**1.4**	**1.8**	**0.8**	**0.1**	**1.5**
	AICD	0.1	-0.5	0.6	0.7	-0.7	2.1
Intracerebral Hemorrhage	Case Count	-0.3	-0.6	0	0	-0.5	0.5
	Case Rate	**-1.7**	**-2.2**	**-1.2**	**-1.8**	**-2.4**	**-1.2**
	Length of Stay	**0.6**	**0.3**	**1.0**	0.3	-0.3	0.8
	AICD	0.2	-0.2	0.7	0.1	-0.6	0.8
Ischemic Stroke	Case Count	-0.1	-0.8	0.6	**-2.0**	**-2.6**	**-1.4**
	Case Rate	**-1.6**	**-2.1**	**-1.1**	**-4.0**	**-4.8**	**-3.2**
	Length of Stay	**-1.2**	**-1.3**	**-1.1**	0.4	0	0.8
	AICD	**-1.3**	**-2.1**	**-0.5**	**-1.6**	**-3.1**	**-0.1**
All Stroke Subtypes	AICD	**-1.0**	**-1.6**	**-0.4**	**-1.2**	**-2.0**	**-0.3**
Stroke Unit Treatment	Case Count	**8.9**	**7.7**	**10.1**	n.a.	n.a.	n.a.
	Case Rate	**7.1**	**5.5**	**8.8**	n.a.	n.a.	n.a.

Statistically significant trends are highlighted in bold. ICD: international classification of diseases; OPS: Operation and Procedure Classification; AAPC: annual average percent change; CI: 95% confidence interval; AICD: annual inpatient case days; n.a.: not applicable (stroke unit treatment is not stratified by patient status at discharge)

**Fig. 1:**
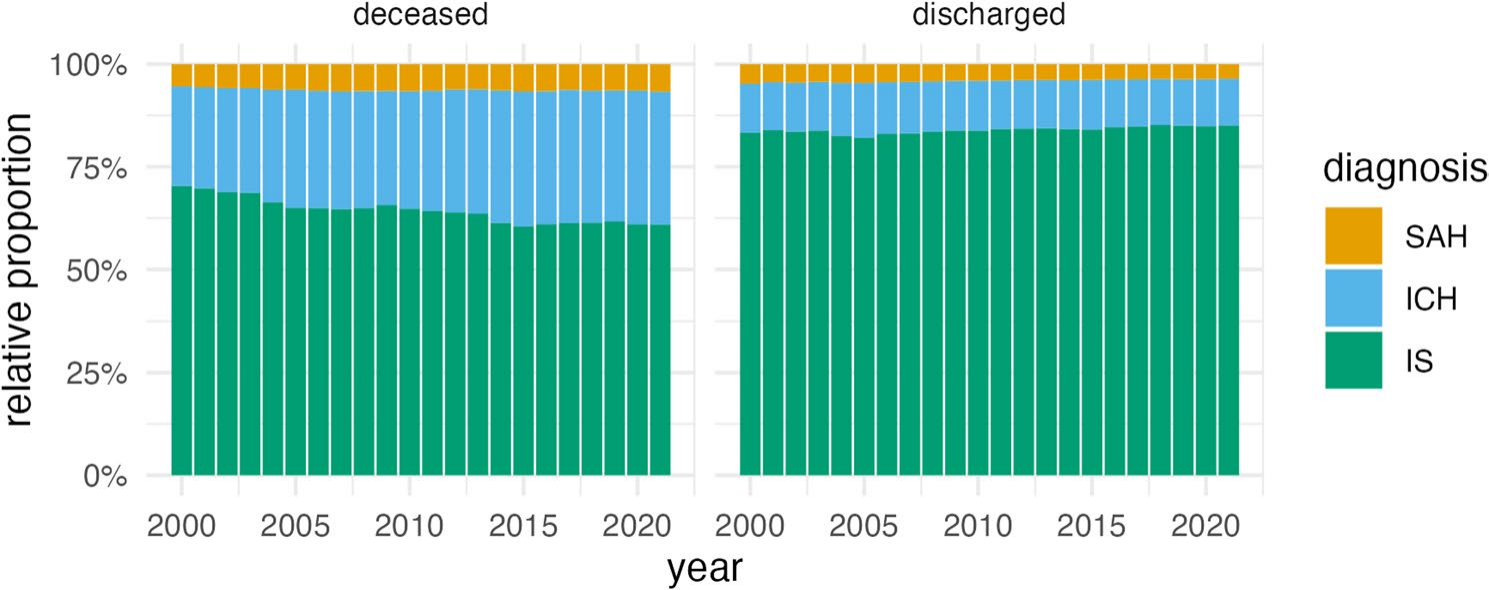
Relative proportions of deceased and discharged cases of stroke subtypes in Germany, 2000–2021. SAH, subarachnoid hemorrhage; ICH, intracerebral hemorrhage; IS ischemic stroke

In 2000, most discharged patients had IS (*n* = 232,780, 83.3%), followed by ICH (*n* = 33,280, 11.9%) and SAH (*n* = 13,345, 4.8%) (Fig. 1). By 2021, the relative proportion of IS slightly increased (*n* = 229,023, 85.1%), while ICH and SAH declined (*n* = 30,705, 11.4% and *n* = 9,558, 3.6%, respectively).

### Trends in stroke subtypes

**Fig. 2:**
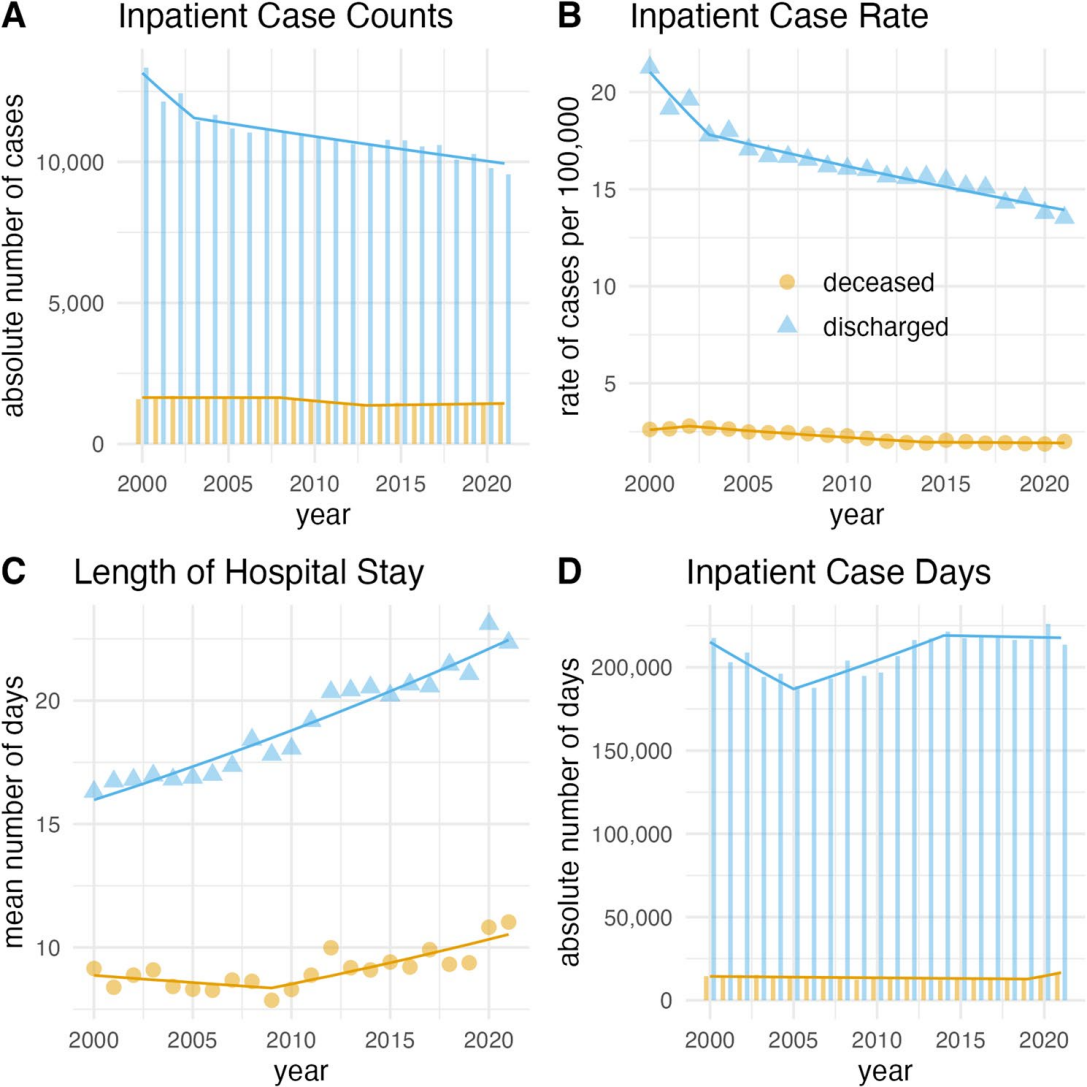
Deceased and discharged cases of subarachnoid hemorrhage in Germany, 2000–2021. Bars, triangles, and points indicate exact values of absolute counts of inpatient cases (**A**), age-standardized rate of inpatient cases per 100,000 people (**B**), mean length of hospital stays (**C**) and the number of annual inpatient case days (**D**). Lines indicate the annual percent change of the respective join point regression model.

For SAH, absolute counts and age-standardized rates declined, whereas mean length of stay increased significantly in both discharged (AAPC = 1.6, 95%-CI 1.4 to 1.8, *p* < 0.001) and deceased cases (AAPC = 0.8, 95%-CI 0.1 to 1.5, *p* = 0.021) (Fig. 2). However, AICD trends were not statistically significant.

**Fig. 3:**
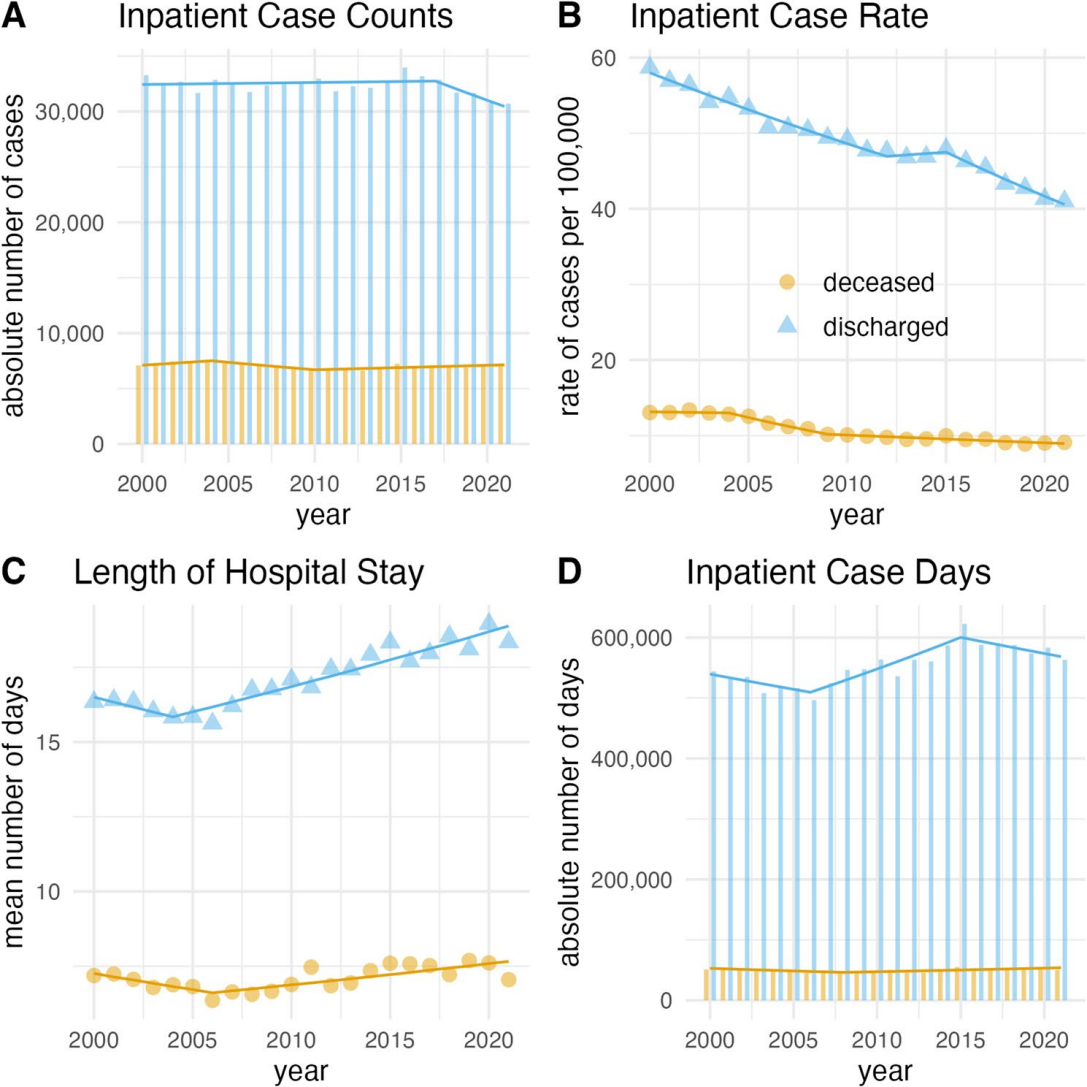
Deceased and discharged cases of intracerebral hemorrhage in Germany, 2000–2021. Bars, triangles, and points indicate exact values of absolute counts of inpatient cases (**A**), age-standardized rate of inpatient cases per 100,000 people (**B**), mean length of hospital stays (**C**) and the number of annual inpatient case days (**D**). Lines indicate the annual percent change of the respective join point regression model.

For ICH, age-standardized rates significantly decreased (discharged: AAPC = -1.7, 95%-CI -2.2 to -1.2, *p* < 0.001; deceased: AAPC = -1.8, 95%-CI -2.4 to -1.2, *p* < 0.001), while absolute counts showed no significant trend. Mean length of stay increased in discharged cases only (AAPC = 0.6, 95%-CI 0.3 to 1.0, *p* < 0.001), without a significant impact on AICD (Fig. 3).

**Fig. 4:**
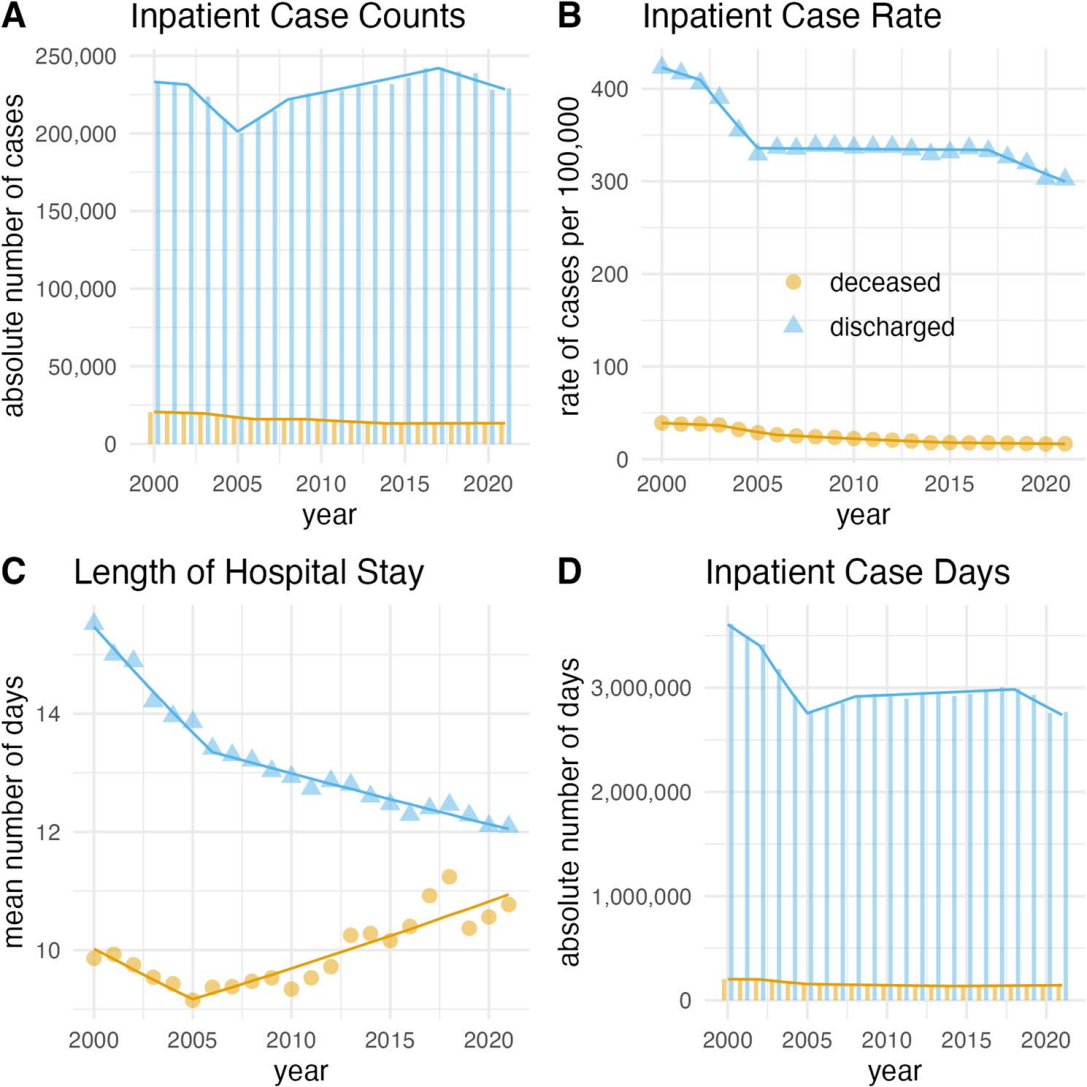
Deceased and discharged cases of ischemic stroke in Germany, 2000–2021. Bars, triangles, and points indicate exact values of absolute counts of inpatient cases (**A**), age-standardized rate of inpatient cases per 100,000 people (**B**), mean length of hospital stays (**C**) and the number of annual inpatient case days (**D**). Lines indicate the annual percent change of the respective join point regression model.

For IS, absolute case counts fluctuated, with a dip in 2005 (*n* = 200,079) and a peak in 2017 (*n* = 242,437), but no significant overall trend. However, age-standardized rates (AAPC = -1.6, 95%-CI -2.1 to -1.1, *p* < 0.001), mean length of stay (AAPC = -1.2, 95%-CI -1.3 to -1.1, *p* = 0.001), and AICD (AAPC = -1.3, 95%-CI -2.1 to -0.5, *p* = 0.002) declined significantly (Fig. 4). A downward trend was evident until 2005/2006, followed by stabilization at remarkably high rates and in length of stay, whereas AICD showed partial reversal. Deceased IS cases were fewer overall, but absolute counts (AAPC = -2.0, 95%-CI -2.6 to -1.4, *p* < 0.001) and age-standardized rates (AAPC = -4.0, 95%-CI -4.8 to -3.2, *p* < 0.001) significantly declined, leading to a decrease in AICD (AAPC = -1.6, 95%-CI -3.1 to -0.1, *p* = 0.04). The proportion of IS coded as ICD-10 I64 declined from 37.1% of discharged cases in 2000 to 0.5% in 2021, with similar patterns in deceased cases.

### Trends in stroke unit treatments

**Fig. 5:**
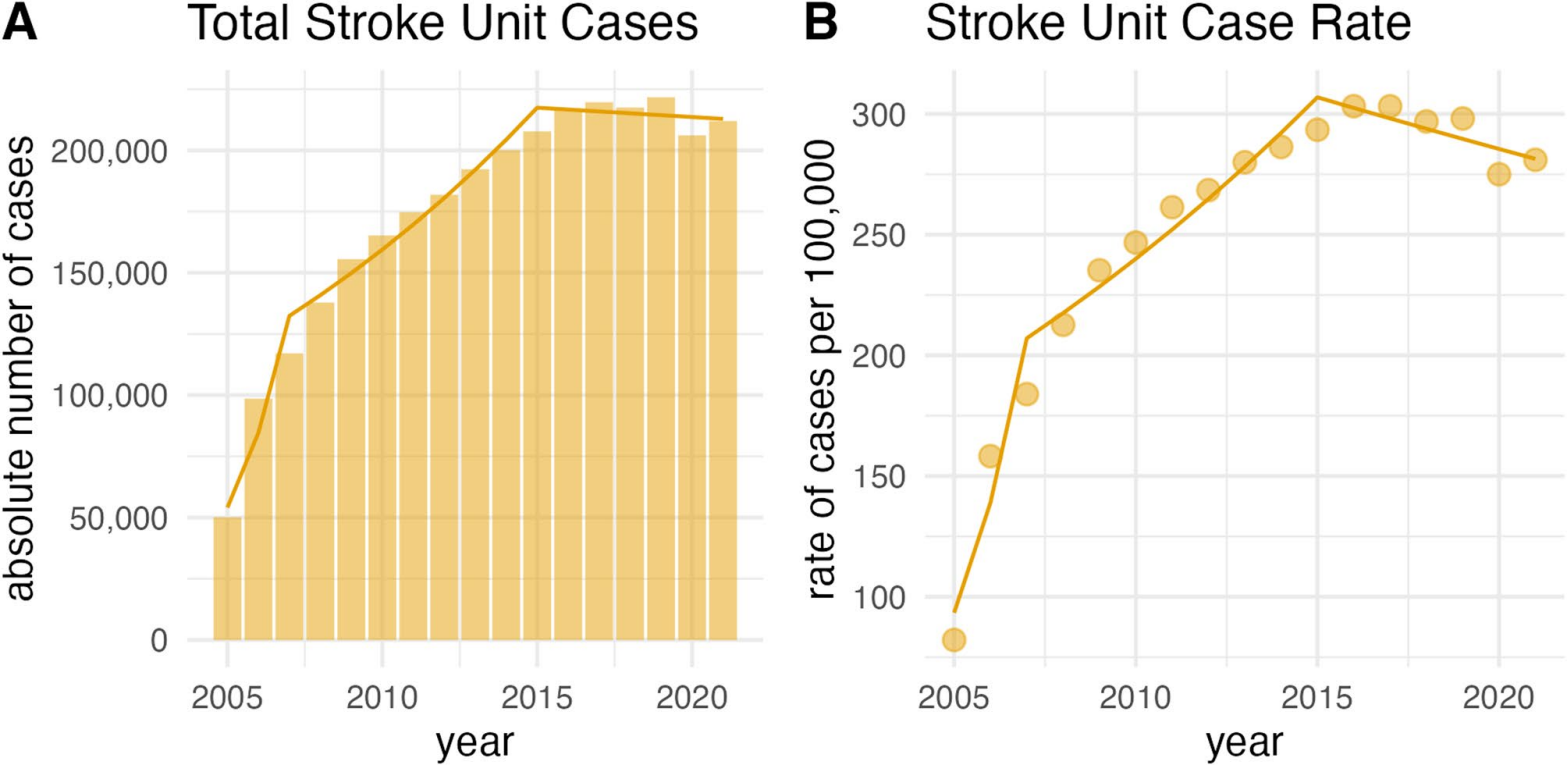
Cases treated in stroke units in Germany, 2005–2021. Bars, triangles, and points indicate exact values of absolute counts of cases (**A**) and age-standardized rate of cases per 100,000 people (**B**). Lines indicate the annual percent change of the respective join point regression model.

From 2005 to 2021, the number of stroke unit (SU)-treated cases increased significantly (Fig. 5). Absolute counts rose by 8.9% annually (95%-CI 7.7 to 10.1, *p* < 0.001), while age-standardized rates increased by 7.1% annually (95%-CI 5.5 to 8.8, *p* < 0.001).

## Discussion

Using hospital administrative data, this study examined long-term trends in acute stroke care in Germany from 2000 to 2021. The overall decline in accumulated annual inpatient case days (AICD) across all stroke subtypes indicates changes in hospitalization patterns and evolving clinical practice.

For IS as the predominant subtype, discharged cases declined strongest until 2005/2006 before stabilizing at a remarkable high level. In contrast, IS-related mortality counts and rates decreased sharply. Between 2000 and 2021, age-standardized mortality-rates decreased by 4% annually on average, extending trends of earlier reported in-hospital mortality reduction from 11.9% in 2005 to 9.5% in 2010 [33]. These findings align with broader improvements in stroke care over the last two decades [34, 35].

The present observations on stroke subtype distribution are in accordance with previous findings, with IS accounting for the majority of cases and a rise in absolute numbers from 2005 to 2017 [36]. Among all subtypes, only IS showed a significant reduction in mean hospital stay among discharged cases. This likely reflects improved treatment efficiency and changes in discharge policies, particularly following the nationwide implementation of the Diagnosis-Related Group payment system in 2005/2006, which incentivized shorter hospital stays [37, 38]. Coding practices also shifted during the study period: IS was increasingly classified under ICD-10 I63 instead of I64, likely due to broader MRI use enhancing diagnostic precision [39]. As pointed out by Nimptsch et al. [33], the 2006 reclassification of “prolonged reversible ischemic neurologic deficit” (PRIND) from G45 (transient ischemic attack) to stroke codes (e.g., I63/I64) likely contributed to rising IS counts.

Our analysis reflects key stages of Germany’s stroke care development. Thrombolysis rates increased markedly from 2005 to 2010 following guideline updates, likely contributing to early mortality improvements [33]. From 2015 onward, mechanical thrombectomy expanded treatment options for patients with large vessel occlusions [40, 41]. While causal inference is limited, these advances, alongside with a numerical increase in SUs likely contributed to the declining in-hospital mortality despite rising IS admissions in some periods.

In a more general perspective, improved acute stroke care may have improved post-stroke survival, increasing the population at risk for recurrent strokes. As seen in other population-based studies, absolute IS case counts rose while age-standardized rates declined, likely driven by demographic aging [42]. Remarkably, the decline in IS admissions and AICD from 2017/2018 onward may partly reflect the impact of reduced hospital admissions during the COVID-19 pandemic [43, 44]. However, recent findings suggest acute stroke care, including mechanical thrombectomy and SU utilization, continued to expand during the pandemic, indicating that these effects may have been transient [41].

In contrast, SAH and ICH demonstrated more consistent declines in age-standardized rates. However, mean hospital stays increased for both discharged and deceased SAH and ICH cases, possibly indicating greater disease complexity, more intensive treatment, or delays in discharge due to limited post-stroke rehabilitation capacity. Persisting SAH and ICH case counts despite falling rates suggest that primary prevention efforts, such as risk factor modification, have not offset the effects of aging population over the study period. Although concerns regarding anticoagulation-related ICH exist [45, 46], no increase in ICH rates was observed, possibly due to the safer profiles of direct oral anticoagulants [47].

The continuous rise in SU-treated patients likely reflects the nationwide expansion of SUs [23, 48] which have been associated with reduced in-hospital mortality [33–35]. Our findings support this, showing mortality declines across all subtypes.

To our knowledge, this is the first study examining acute stroke care trends in Germany over such an extended period. Nevertheless, several limitations apply. Secondary administrative data do not capture pre-hospital mortality, and data quality may vary across hospitals. The case-based design prevents tracking individuals or distinguishing first from recurrent events. Observed trends may reflect administrative changes (e.g., ICD coding shifts) as much as clinical practice. Upcoding due to financial incentives may also have biased results [49]. Furthermore, the analysis was limited to discharge status, without adjustment for individual-level variables such as age, sex, or socioeconomic status, which should be addressed in future work [50–54].

Despite these limitations, our findings may inform future research on stroke care trends and support planning efforts to meet the needs of an aging population.

## Conclusions

This study provided a comprehensive analysis of acute stroke care trends in Germany from 2000 to 2021, highlighting subtype-specific patterns. The overall inpatient burden declined, primarily driven by reductions in IS admissions and mortality. However, the number of IS cases remains at a remarkably high level along with a rising mean length of hospital stays for SAH and ICH and an overall increasing SU care, indicating shifting but consistently high resource requirements. Thus, our findings underline the continued need to optimize healthcare infrastructure. In addition, future planning should consider a subtype-specific resource allocation in acute stroke care.

## Data Availability

Publicly available datasets were used in this study (Data source: Statistisches Bundesamt (Destatis), Genesis-Online; Data licence by-2-0). Calculations will be provided by the corresponding author upon request.

## Abbreviations

AAPC: Average annual percent change
AICD: Annual inpatient case days
APC: Annual percent change
CI: Confidence intervals
ICH: Intracerebral hemorrhage
IS: Ischemic stroke
JPR: Joinpoint regression
OPS: German operation and procedure classification
SAB: Subarachnoid hemorrhage
SU: Stroke unit
PRIND: Prolonged reversible ischemic neurologic deficit
